# The interaction between stress and positive affect in predicting mortality

**DOI:** 10.1016/j.jpsychores.2017.07.005

**Published:** 2017-09

**Authors:** Judith A. Okely, Alexander Weiss, Catharine R. Gale

**Affiliations:** a Centre for Cognitive Ageing and Cognitive Epidemiology, Department of Psychology, University of Edinburgh, Edinburgh, UK; b Department of Psychology, University of Edinburgh, Edinburgh, UK; c MRC Lifecourse Epidemiology Unit, University of Southampton, Southampton, UK

**Keywords:** Positive emotions, Stress and coping measures, Mortality, Longitudinal study

## Abstract

**Objective:**

Positive affect is associated with longevity; according to the stress-buffering hypothesis, this is because positive affect reduces the health harming effects of psychological stress. If this mechanism plays a role, then the association between positive affect and mortality risk should be most apparent among individuals who report higher stress. Here, we test this hypothesis.

**Methods:**

The sample consisted of 8542 participants aged 32–86 from the National Health and Nutrition Examination Survey (NHANES I) Epidemiological Follow-up Study (NHEFS). We used Cox's proportional hazards regression to test for the main effects of and the interaction between positive affect and perceived stress in predicting mortality risk over a 10 year follow up period.

**Results:**

Greater positive affect was associated with lower mortality risk. We found a significant interaction between positive affect and perceived stress such that the association between positive affect and mortality risk was stronger in people reporting higher stress. In the fully adjusted model, a standard deviation increase in positive affect was associated with a 16% (HR = 0.84; 95% CI = 0.75, 0.95) reduction in mortality risk among participants who reported high levels of stress. The association between positive affect and mortality risk was weaker and not significant among participants who reported low levels of stress (HR = 0.98; 95% CI = 0.89, 1.08).

**Conclusion:**

Our results support the stress-buffering model and illustrate that the association between positive affect and reduced risk may be strongest under challenging circumstances.

## Highlights

• The inverse association between positive affect and mortality risk is stronger in people reporting higher subjective stress.
• This finding supports the stress buffering model of positive affect and health
• Individual differences in positive affect may be most salient under challenging circumstances

## 1 Introduction

Positive affect is a component of psychological wellbeing and can be defined as the experience of positive emotion such as happiness, joy, excitement, or contentment [1]. People who experience frequent positive affect tend to live longer, healthier lives [2], [3], [4]. The discovery that this association is not fully explained by differences in demographic factors or depressive symptoms has led authors to suggest that positive affect is causally related to physical health [2], [3], [4]. However, the mechanisms by which positive affect impacts health outcomes is not fully understood. Pressman and Cohen [1] proposed two potentially compatible models that could explain this association. According to the direct effects model, the experience of positive affect impacts directly on physiological processes and health behaviours associated with healthy functioning. The stress-buffering model, on the other hand, proposes that positive affect is associated with good health because it protects against the pathogenic consequences of psychological stress [1]. If the positive association between positive affect and better health is caused by this stress buffering mechanism, then the protective effect of positive emotion should be stronger for people who experience more stress. In other words, psychological stress should moderate the association between positive affect and health. To date, researchers interested in the link between higher positive affect and lower mortality risk have focused on the direct effects model; consequently, it is unclear whether perceived stress moderates this risk association.

Positive affect can be measured at the trait or state level; trait measures assess how an individual ‘typically’ feels and state measures assess how an individual feels at a particular point in time. Both trait and state measures of positive affect have been linked to longevity [4] and biomarkers of neuroendocrine, inflammatory and cardiovascular functioning [1], [5], [6]. There is evidence that positive and negative affect represent independent constructs rather than opposite points on a continuum and that these construct are independently associated with mortality risk [2], [7], [8].

The idea that positive affect serves an adaptive function during periods of stress was prompted by the observation that stress and positive affect can co-occur [9]. For example, in a longitudinal study of 253 male caregivers, participants reported experiencing positive affect as frequently as they did negative affect [10]. Accounts of positive affect during periods of severe stress can also be found in studies into the process of bereavement [11], [12], and the onset of disability [12].

Pressman and Cohen [1], hypothesize that the experience of positive affect during periods of stress could reduce behavioural and physiological stress responses. Health harming responses to stress include overactivation of allostatic systems, such as the hypothalamic–pituitary–adrenal (HPA) axis or the autonomic nervous system (ANS) [13], and an increase in unhealthy behaviours such as smoking, alcohol consumption, or substance abuse [14]. The stress buffering model identifies physiological and psychosocial factors associated with positive affect that may interact with these stress responses [1]. Firstly, at a physiological level, the release of endogenous opioids (a correlate of high positive affect) could dampen HPA and ANS responses to stress [15], [16]. At a cognitive level, positive affect may facilitate creative problem solving or the appraisal of a stressful situation as an opportunity or challenge [17], [18]. These responses may reduce exposure to stressors, and, consequently, both HPA and ANS activity, as well as health harming behaviours. Finally, Pressman and Cohen [1] suggest that individuals who experience more positive affect are more likely to have social and physical resources that facilitate adaptive coping—both at a behavioural and physiological level. Similar mechanisms are proposed in Fredrickson's Broaden-and-Build theory [19], [20], which posits that the experience of positive affect can help individuals build the psychosocial resources needed to cope with stress and adversity. Fredrickson [19] also proposes that the experience of positive emotions following a stressful experience can help undo the physiological responses (specifically cardiovascular reactivity) and cognitive responses (narrowing of the thought-action repertoire) to stress [19].

Studies of positive affect and stress responses provide evidence for the mechanisms identified in the stress-buffering model and the Broaden-and-Build theory [19]. Several studies have tested whether positive affect dampens physiological responses to laboratory stress tasks. Fredrickson, Mancuso, Branigan, and Tugade [20] measured cardiovascular recovery following a stress induction task in 170 students. Participants who viewed films that elicited amusement or contentment following the stress task were characterized by quicker cardiovascular recovery than participants who viewed neutral films or films that elicited sadness. Similarly, in 170 participants, Kraft and Pressman [21] found that maintaining a positive (versus neutral) facial expression during a stress task was associated with lower heart rate during the stress recovery period. Finally, in 72 healthy men, frequency of self-reported positive affect was associated with lower systolic blood pressure during a stress task and quicker diastolic pressure recovery following the task [5]. Although less is known regarding associations between stress, positive affect, and health behaviours, there is evidence that greater wellbeing is associated with positive behaviour change following stressful events, such as diagnosis of chronic disease [22], [23], [24]. In addition, in a longitudinal study of 83 college students, positive affect was associated with better sleep efficiency (hours of sleep/time in bed) on days of higher stress but not on days of lower stress [25].

Fewer studies have tested the key prediction from these theories, that is, there should be an interaction between positive affect and perceived stress in predicting health outcomes. In a cross-sectional study of 382 participants, the association between higher stress and lower self-rated health was significantly moderated by positive affect such that the association was strongest at low levels of positive affect [26]. Blevins, Sagui, and Bennett [27] tested whether self-reported stress moderated the association between higher positive affect and lower levels of systemic inflammation. Using cross-sectional data from the National Longitudinal Study of Adolescent to Adult Health (n = 3093), they found that higher positive affect was associated with lower levels of inflammation only among participants who reported higher levels of stress. Finally, in an experimental study (n = 60), Robles, Brooks, and Pressman [28] compared the strength of the association between positive affect and skin barrier recovery (following a ‘tape stripping’ procedure) between participants assigned to a stress condition and participants assigned to a control condition. Higher positive affect was associated with faster recovery in the stress condition but not in the control condition.

In a recent meta-analysis on positive affect as a predictor of longevity, Zhang and Han [4] identified one study that tested for an interaction between perceived stress and positive affect. This study used data from the National Health and Nutrition Examination Study I (NHANES I) Epidemiologic Follow-Up Study (NHEFS) [29]. The authors found evidence of a stress-buffering effect only in a subsample of participants who had no chronic conditions and were over the age of 65. In this subsample, the association between higher positive affect and lower mortality risk was strongest among participants that reported higher stress. However, as the primary aim of Moskowitz and colleagues' [29] study was to compare participants with and without diabetes, the sample was restricted to participants diagnosed with diabetes (n = 715) and participants without any chronic conditions (n = 2673).

In summary, previous studies report that positive affect protects against some health harming responses to stress and that positive associations between positive affect and better health are stronger under conditions of high stress. However, it is unclear whether this moderating effect applies to the association between higher positive affect and lower mortality risk. In the current study, we tested whether perceived stress moderated the positive association between positive affect and longevity in a large, nationally representative sample.

## 2 Methods

### 2.1 Participants

We used data from the NHEFS [30]. The NHANES I (1971–1975) data were taken from a nationwide probability sample of 32,000 Americans aged 1 to 74. The NHEFS began in 1982 and included 12,220 participants aged 25–74 who had completed the medical examination in NHANES I. Subsequent waves of NHEFS data collection were conducted in 1986, 1987, and 1992. Of the 12,220 participants in the NHEFS sample, we excluded 1697 participants due to missing vital status data and an additional 1,981 participants due to missing covariate data. This left us with an analytic sample of 8542 participants. The excluded participants differed from the analytic sample on several variables (see Supplementary Table 1 for a summary of these differences).

### 2.2 Measures

Positive affect, stress, and covariate measures, apart from wealth and height, were taken from the NHEFS wave 1 (1982) interview. Wealth and height were taken from the NHANES I (1971–1975) interview.

#### 2.2.1 Positive affect

As has been done previously [31], [32], positive affect was measured using the positive affect subscale of the General Wellbeing Questionnaire (GWQ) [33]. The positive affect subscale consists of three questions: “How have you been feeling in general in the past month?” (anchors were “in excellent spirits” and “in very low spirits”), “How happy, satisfied, or pleased have you been with your personal life, during the past month?”, and “How much energy, pep, vitality have you felt, during the past month?”. This subscale's scores range from 0 to 20 with higher scores indicating higher positive affect. Cronbach's alpha for this scale in our sample was 0.60.

#### 2.2.2 Stress

Following Moskowitz et al. [29], we used three items from the GWQ as a measure of perceived stress. The items were: “Have you been under or felt you were under any strain, stress, or pressure during the past month?”, “Have you been anxious, worried or upset, during the past month?”, and “How relaxed or tense have you been during the past month?”. Scores ranged from 1 to 22 with higher scores indicating higher levels of perceived stress. Cronbach's alpha for this scale in our sample was 0.75. There is no clear agreement on the definition of perceived stress in the literature [34]; however, the stress items used in our study are comparable to a subset of those used for the stress scale of the Depression Anxiety Stress Scales (DASS) [35], [36], a popular measure of subjective stress. Similar DASS items include: “I found it difficult to relax”, “I found it hard to wind down”, “I was in a state of nervous tension”, and “I found myself getting upset rather easily”. The DASS defines depressive symptoms in terms of low mood, motivation, and self-esteem and stress in terms of tension, nervousness and irritability [35], [36].

#### 2.2.3 Mortality

Participants' vital status was recorded until the end of 1992. Information regarding date and cause of death was obtained from death certificates.

#### 2.2.4 Covariates

We adjusted for variables that might confound or mediate the association between positive affect and mortality risk. These included age, sex, race/ethnicity, socio-economic status, level of education, depressive symptoms, marital status, physical activity, smoking status, fruit and vegetable consumption, alcohol consumption, body mass index (BMI), sleep duration, and history of cancer, cardiovascular disease, and chronic lung disease. These factors have previously been associated with mortality risk [37], [38], [39], [40], [41], [42], [43], [44], [45], [46], [47], [48], [49], [50] as well as positive affect or wellbeing [1], [41], [51], [52], [53], [54], [55], [56], [57], [58].

Wealth was indexed by family income from all sources over the past 12 months. We chose to use the family income measure from the NHANES 1 (1971–1975) interview rather than NHEFS (1982) interview as the later had a larger amount of missing data (n = 828). Family income measures from NHANES 1 and NHEFS were strongly correlated (r = 0.66). Responses to the family income question in NHANES 1 were recorded as either less than $1000, a specific quantity between $1000 and $25,000, or $25,000 or more. We grouped participants into four income categories: <$3000, $3000–$5999, $6000–14,999 and >$14,999. Education was measured as the highest year of regular school (including college) attended. Based on their responses, we grouped participants into 4 categories: ≤ 8 years of education, 9–11 years, 12 years, and > 12 years. Based on the information available, participants' race/ethnicity was categorized as “black”, “other”, or “white”. Depressive symptoms were assessed using the Center for Epidemiologic Studies Depression Scale (CES-D) [59]. The CES-D consists of twenty items and is designed to measure symptoms of depression in the general population. The CES-D score was treated as a continuous variable. Participants reported whether they were married, divorced, widowed, or never married. We used these responses to create two categories: “married” and or “not married”. Participants were asked to report the amount of physical activity they engaged in during recreational activities and during a typical day (excluding recreational physical activity). Response options were “much exercise”, “moderate exercise”, and “little or no exercise”. As responses to these two questions about physical activity were distributed differently, we created two separate variables: recreational physical activity and non-recreational physical activity. Participants were asked whether they had ever smoked > 100 cigarettes and whether they were a current smoker. Based on response to these two questions, participants were classified as non-smokers, former smokers, and current smokers. Participants were asked to estimate the number of servings of fruit and vegetables they had per day. We dichotomized responses based on number of servings – either 5 or more servings per day or < 5 servings per day. Participants were asked to describe their drinking habits using the response options “abstainer”, “light drinker”, “moderate drinker”, and “heavy drinker”. As only 69 participants identified themselves as heavy drinkers, we grouped heavy and moderate drinkers in the same category. Participants were asked to estimate the average number of hours they slept each night. As has been done previously [60], we categorized sleep duration as 4 h or fewer, between 5 and 9 h, and 10 or more hours. Although participants' weight was measured in 1982, height measures were only taken for NHANES I (1971–1975). We thus computed participant BMI from these two measures and treated BMI as a continuous variable. The correlation between the 1971–1975 height and weight measures (r = 0.47, p < 0.001) was not significantly different from the correlation between the 1971–1975 height measure and the 1982 weight measure (r = 0.48, p < 0.001) (z = 1.46, p = 0.07). Finally, participants were asked if a doctor had ever diagnosed them with cancer (breast cancer, skin cancer, or any other type of cancer), cardiovascular disease (CVD) (stroke or heart attack), or chronic lung disease (chronic bronchitis or emphysema).

### 2.3 Statistical analysis

Cox's proportional hazard regressions were used to examine the association between positive affect and perceived stress at baseline and mortality over the follow-up period. Survival time in days was calculated from the date of the first NHEFS interview to the date of death or, for participants who did not die during the follow up, the date of their last interview.

We adjusted for covariates in three stages. Model 1 was adjusted for age and sex. Model 2 was additionally adjusted for potentially confounding variables: demographic variables (race/ethnicity, wealth, education, marital status), history of chronic conditions (cancer, CVD or respiratory disease), and depressive symptoms. Model 3 was further adjusted for potentially mediating variables: health behaviours (smoking status, alcohol consumption, physical activity, and diet), sleep duration, and BMI.

We tested whether the association between positive affect and mortality risk varied per level of stress by including a stress score × positive affect score interaction term in each of the three models.

## 3 Results

Table 1 shows the baseline characteristics of the sample (n = 8542) according to positive affect tertile. On average, participants with higher positive affect were more likely to be male, younger, and married. These participants also tended to be wealthier, better educated, and engaged in more recreational and non-recreational physical activities, and had fewer depressive symptoms, lower perceived stress, and a lower BMI. Finally, on average, participants with higher positive affect consumed more alcohol, ate more fruits and vegetables, were less likely to sleep < 5 h or > 9 h a night, and were less likely to report a history of chronic disease. Supplementary Table 2 shows correlations among positive affect, perceived stress, and the other baseline characteristics. The correlation between positive affect and perceived stress score was r = − 0.51 (p < 0.001).

**Table 1:** Baseline characteristics stratified according to tertiles of positive affect score (low, moderate and high positive affect)^a^ total n = 8542.

Characteristics	Low	Moderate	High	p-Trend^b^
Age *M* (SD)	57.85 (14.97)	55.66 (14.32)	53.68 (13.64)	< 0.001
Female, no. (%)	2095 (69)	1977 (64)	1299 (54)	< 0.001
Race/ethnicity no. (%)				0.46
Black	389 (13)	361 (12)	282 (12)	
White	2643 (87)	2713 (87)	2069 (87)	
Other	20 (1)	28 (1)	37 (2)	
Married, no. (%)	1936 (63)	2208 (71)	1809 (76)	< 0.001
Wealth category $, no. (%)^c^				< 0.001
< 3000	438 (14)	275 (9)	174 (7)	
3000–5999	584 (19)	483 (16)	318 (13)	
6000–14,999	1504 (49)	1570 (51)	1172 (49)	
> 14,999	526 (17)	774 (25)	724 (30)	
Education category, no. (%)				< 0.001
≤ 8 years	767 (25)	590 (19)	404 (17)	
9–11 years	575 (19)	488 (16)	340 (14)	
12 years	1099 (36)	1211 (39)	878 (37)	
> 12 years	611 (20)	813 (26)	766 (32)	
CESD score *Mdn* (IQR)	12 (6–18)	5 (2–10)	3 (0–6)	< 0.001
Stress score *M* (SD)	10.26 (4.68)	7.55 (3.90)	5.33 (3.35)	< 0.001
BMI (kg/m^2^) *M* (SD)	26.50 (5.38)	26.36 (4.95)	25.93 (4.38)	< 0.001
Recreational activity, no. (%)				< 0.001
Inactive	1443 (47)	975 (31)	497 (21)	
Moderate	1336 (44)	1652 (53)	1260 (53)	
Vigorous	273 (9)	475 (15)	631 (26)	
Non-recreational activity, no. (%)				
Inactive	786 (26)	408 (13)	201 (08)	
Moderate	1746 (57)	1841 (59)	1205 (50)	
Vigorous	520 (17)	853 (27)	982 (41)	
Alcohol consumption, no. (%)				< 0.001
Abstainer	1498 (49)	1304 (42)	886 (37)	
Light drinker	1249 (41)	1419 (46)	1222 (51)	
Moderate drinker	305 (10)	379 (12)	280 (12)	
Smoking status, no. (%)				0.64
Non-smoker	1370 (45)	1447 (47)	1056 (440	
Former smoker	792 (26)	826 (27)	669 (28)	
Smoker	890 (29)	829 (27)	663 (28)	
≥ 5 servings of fruit and veg, no. (%)	1049 (34)	1191 (38)	922 (39)	0.001
Sleep duration categories, no. (%)				< 0.001
< 5 h	125 (4)	51 (2)	30 (1)	
5–9 h	2810 (92)	2959 (95)	2307 (97)	
> 9 h	117 (4)	92 (3)	51 (2)	
History of CVD, no. (%)	274 (9)	161 (5)	72 (3)	< 0.001
History of cancer, no. (%)	309 (10)	258 (8)	182 (8)	0.003
History of chronic lung disease, no. (%)	443 (15)	282 (9)	135 (6)	< 0.001

a The cut points for positive affect tertiles were based on the analytic sample.

b Statistical significance is based χ^2^ tests or one-way ANOVA, as appropriate.

c $3000 in 1975 has the equivalent value of $13,646 in 2017.

Over the 10-year follow-up period, 1507 deaths were reported. Supplementary Table 3 shows bivariate associations between positive affect, perceived stress or covariate variables, and mortality risk.

In a model adjusted for age, sex, perceived stress, and positive affect, positive affect was associated with lower mortality risk (hazard ratio [HR]: 79; 95% confidence interval [CI]: 0.74–0.84) and stress was not associated with mortality risk (HR: 0.97; 95% CI: 0.91–1.03). We re-ran this model additionally including the interaction effect between positive affect and perceived stress. The interaction between positive affect and perceived stress was significant in the age- and sex-adjusted model (p < 0.001) and remained significant following adjustment for demographic variables, history of chronic disease and depressive symptoms (model 2) (p = 0.02), and health behaviours, and BMI (model 3) (p = 0.04). Table 2 displays the results of this fully adjusted model.

**Table 2:** HRs (95% CI) for all-cause mortality for variables in the fully adjusted model testing for a positive affect × stress interaction.

Variable	HR (95% CI)	p
Positive affect	0.91 (0.85–0.97)	0.005
CESD	1.00 (1.00–1.01)	0.24
Stress	0.90 (0.84–0.97)	0.008
Age	1.09 (1.08–1.09)	< 0.001
BMI	0.99 (0.98–1.00)	0.041
Sex: male vs. female	1.82 (1.61–2.07)	< 0.001
Marital status: married vs. single	0.77 (0.69–0.87)	< 0.001
Race/ethnicity		
Black vs. white	1.11 (0.95–1.30)	0.19
Other ethnicity vs., white	1.15 (0.66–2.00)	0.61
Wealth $		
3000–5999 vs. < 3000	0.96 (0.83–1.12)	0.61
6000–14,999 vs. < 3000	1.01 (0.87–1.18)	0.89
> 14,999 vs. < 3000	0.90 (0.72–1.11)	0.31
Education		
9–11 years vs. ≤ 8 years	0.97 (0.83–1.13)	0.69
12 years vs. ≤ 8 years	0.87 (0.75–1.00)	0.052
> 12 years vs. ≤ 8 years	0.80 (0.68–0.95)	0.013
Recreational activity		
Moderate vs. inactive	0.87 (0.77–0.99)	0.031
Vigorous vs. inactive	0.75 (0.61–0.91)	0.003
Non-recreational activity		
Moderate vs. inactive	0.75 (0.65–0.86)	< 0.001
Vigorous vs. inactive	0.67 (0.56–0.80)	< 0.001
Alcohol consumption		
Light drinker vs. abstainer	0.90 (0.80–1.012)	0.074
Moderate drinker vs. abstainer	1.06 (0.86–1.30)	0.60
Smoking status		
Former smoker vs. non-smoker	1.23 (1.08–1.40)	0.001
Smoker vs. non-smoker	1.65 (1.42–1.92)	< 0.001
Diet: ≥ 5 fruit and vegetables vs. < 5	0.98 (0.88–1.09)	0.71
Sleep duration		
5–9 h vs. < 5 h	0.86 (0.65–1.14)	0.30
> 9 h vs. < 5 h	0.96 (0.68–1.34)	0.79
History of CVD vs. no history	1.75 (1.52–2.02)	< 0.001
History of cancer vs. no history	1.52 (1.33–1.74)	< 0.001
History of chronic lung disease vs. no history	1.27 (1.10–1.46)	0.001
Positive affect × stress	1.04 (1.00–1.09)	0.036

To facilitate interpretation of the interaction effect, we divided the sample into tertiles according to perceived stress (low, moderate, and high), and conducted an analysis for each group separately. In the age- and sex-adjusted model, higher positive affect was associated with a lower mortality risk in all three groups. However, a stronger effect was observed in the higher perceived stress groups; a standard deviation (SD) increase in positive affect score was associated with a 13% reduction in mortality risk (HR: 87; 95% CI: 0.80–0.94) in the low perceived stress group, a 24% (HR: 0.76; 95% CI: 0.67–0.86) reduction in the moderate perceived stress group, and a 31% (HR: 0.69; 95% CI: 0.63–0.76) reduction in the high perceived stress group. In model 2, the association between positive affect and mortality risk remained significant, although it was attenuated for all three groups. Again, a stronger effect was observed in the group with the highest levels of perceived stress; HRs for low, moderate, and high perceived stress groups per SD increase in positive affect score were 0.90 (95% CI: 0.82–0.98), 0.82 (95% CI: 0.72–0.93), and 0.77 (95% CI: 0.68–0.86), respectively. In the fully adjusted model (model 3), the association between positive affect and mortality risk was significant in the moderate (HR: 0.85; 95% CI: 0.74–0.97) and high (0.84; 95% CI: 0.75–0.95) perceived stress groups, but not in the low perceived stress group (HR: 0.98; 95% CI: 0.89–1.08). Table 3 displays HRs for all-cause mortality for each SD increase in positive affect.

**Table 3:** HRs (95% CIs) for all-cause mortality according to a SD increase in positive affect score divided by tertiles of perceived stress score.

	Cases/N	Model 1	Model 2	Model 3
Low stress	665/2996	0.87 (0.80–0.94)⁎⁎	0.90 (0.82–0.98)⁎	0.98 (0.89–1.08)
Moderate	452/2807	0.76 (0.67–0.86)⁎⁎	0.82 (0.72–0.93)⁎⁎	0.85 (0.74–0.97)⁎
High stress	390/2739	0.69 (0.63–0.76)⁎⁎	0.77 (0.68–0.86)⁎⁎	0.84 (0.75–0.95)⁎⁎

Model 1: Adjusted for age and sex. Model 2 Further adjusted for demographic factors, history of chronic disease and depressive symptoms. Model 3 additionally adjusted for health behaviours, sleep duration and BMI.

⁎⁎ p < 0.001.

⁎ p < 0.05.

**Fig. 1:**
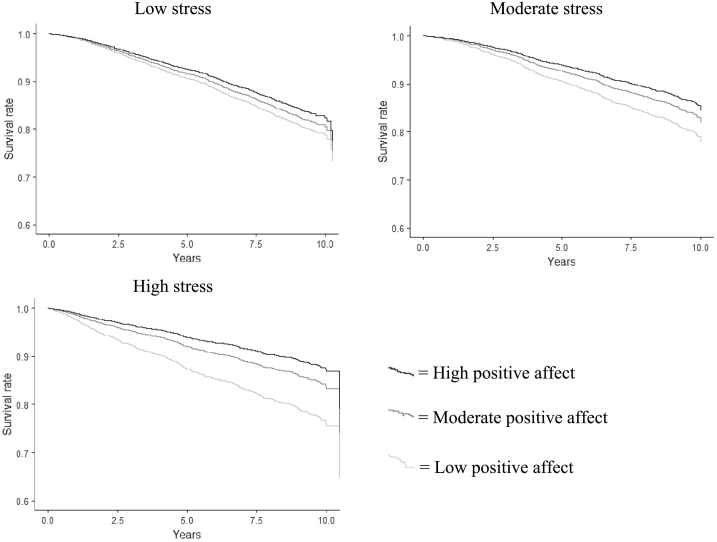
Survival probabilities (adjusted for age and sex) for the low, moderate and high stress groups stratified by tertile of positive affect.

Fig. 1 displays survival probabilities for the low, moderate, and high perceived stress groups stratified by tertile of positive affect. It should be noted that the association between perceived stress group and mortality risk was different from the association between perceived stress score (which was treated as a continuous variable) and mortality risk. Following adjustment for age, sex and positive affect tertile, participants in the moderate stress group had a lower mortality risk than participants in the low stress group. Mortality risk for participants in the low and high stress groups were not significantly different.

### 3.1 Additional analysis

We ran additional analysis to examine the association between stress and mortality risk. Following adjustment for age and sex, higher perceived stress was associated with a higher mortality risk (HR: 1.10; 95% CI: 1.04–1.16). This association was not significant following additional adjustment for demographic differences, depressive symptoms, history of chronic disease, and health behaviours (HR: 0.95; 95% CI: 0.89–1.02). However, when we additionally adjusted for positive affect, the association between stress and mortality risk became inverse and significant (HR: 0.93; 95% CI: 0.86–1.00). This inverse association was still significant after we included the interaction between perceived stress and positive affect. See Table 2 for the results of this fully adjusted model.

We calculated the impact of adjusting for health behaviours or wealth and education on the HR for the interaction between positive affect and perceived stress. We achieved this using the formula ([HR adjusted for age and sex − 1] − [HR adjusted for age, sex and covariate − 1] / [HR adjusted for age − 1]) × 100 [61]. Adjusting for health behaviours attenuated the HR by 19%, adjusting for wealth and education attenuated the HR by 11%. We additionally tested if the interaction between perceived stress and positive affect varied as a function of wealth or education by including a three-way interaction term between perceived stress, positive affect and wealth or education. These three-way interaction effects were not significant (p = 0.42 for wealth, p = 0.73 for education).

The positive affect measure had a relatively low alpha (α = 0.60). This was improved by excluding the vitality item (“How much energy, pep, vitality have you felt during the past month?”) (α = 0.71). In addition, there is evidence that the subdomain of energy or vitality may underlie positive associations between positive affect and longevity [1]. To examine the effect of the vitality item, we re-ran all analyses after excluding this item from the positive affect measure. The interaction between perceived stress and positive affect was significant in the age- and sex-adjusted model (p = 0.001) and in the model additionally adjusted for demographic differences (p = 0.045), but not in the fully adjusted model (p = 0.059). Positive affect was not associated with mortality risk in any of the perceived stress tertiles in the fully adjusted model. See Supplementary Table 4 for a summary of these results.

There are two separate measures of positive affect in the NHEFS study: the GWQ positive affect measure and CES-D positive affect subscale. To test whether we would find similar results with a different positive affect measure, we re-ran the analysis replacing the GWQ positive affect measure with the CES-D subscale. The interaction between perceived stress and positive affect was significant in the age- and sex-adjusted model (p = 0.017), but not in the model additionally adjusted for demographic factors (p = 0.065) or in the fully adjusted model (p = 0.078).

There is evidence that subdomains of depressive symptoms are differentially associated with health behaviours [62]. The CES-D can be divided into subdomains of negative affect, anhedonia and somatic symptoms [63]. To specifically test for the role of negative affect, we re-ran the analysis replacing CES-D with negative affect. As was the case for CES-D, negative affect was not a significant predictor of mortality risk in the fully adjusted model (see Table 3). The results for positive affect were very similar to those in our original analysis. In the fully adjusted model, HRs for positive affect in the moderate and high perceived stress tertiles were 0.01 unit lower than in the original analysis.

Proportional hazard assumptions were not met for age, history of cancer and BMI. To address this violation of the proportionality assumption, we re-ran the fully adjusted model using the step-approach method [64]. This approach allowed us to model the change in the effect of age, history of cancer and BMI over time. HRs for positive affect, perceived stress and the positive affect × perceived stress interaction were the same as in the original fully adjusted model.

## 4 Discussion

According to the stress buffering model, positive affect may protect against some health harming consequences of psychological stress [1]. The link between higher positive affect and longevity may therefore be most pronounced among individuals who experience stress. In this large nationally-representative sample, we found a significant interaction between perceived stress and positive affect; the association between higher positive affect and longevity was strongest among participants who reported higher stress. This interaction remained significant following adjustment for depressive symptoms, demographic factors, history of chronic disease, and health behaviours. The strength of the association between positive affect and mortality risk was similar (and statistically significant) in the fully adjusted model for participants who reported moderate and high levels of stress. This finding suggests that even individuals with moderately elevated stress may benefit from positive affect.

There are various mechanisms that might account for the stress-buffering effect; positive affect may lessen HPA and ANS activity as well as health harming behavioural responses to stress [1]. Although we were unable to test for physiological responses to perceived stress in our study, we did examine the effect of health behaviours. Adjusting for health behaviours attenuated the interaction effect between positive affect and perceived stress by 19%—suggesting that the stress buffering effect is partially explained by differential behavioural responses to stress. Specifically, individuals with high positive affect may be less likely to engage in health harming behaviours during periods of stress. Pressman and Cohen [1] further suggest that positive affect may reduce the experience of or exposure to psychological stress. This idea was supported by the strong negative correlation between positive affect and perceived stress in our study. However, as this association was cross-sectional, the direction of the relationship between perceived stress and positive affect is unclear.

It is also possible that the interaction between positive affect and stress is confounded by SES. Individuals with higher positive affect in our study also tended to have more wealth and more years of education. Prior research has identified SES as a key modifier of the association between stress and mortality risk; stress is most strongly associated with mortality risk in low SES groups [65]. We found some evidence of a confounding effect in our study. Adjusting for wealth and education attenuated the interaction between perceived stress and positive affect by 11%.

The pattern of results in our study is similar to those of two cross-sectional studies. Specifically, Blevins et al. [27] and Bränström [26] found that the associations between higher positive affect and lower levels of inflammatory markers and better self-rated health, respectively, were stronger among participants who reported higher levels of stress. Our study builds on previous findings by demonstrating that a stress-buffering effect can be found in longitudinal data and for all-cause mortality risk. In addition, these data allowed us to adjust for potentially mediating or confounding variables, including physical activity, alcohol consumption, and diet, that were not included in previous cross-sectional studies.

Our results partially contrast with those reported by Moskowitz et al. [29] who found that the association between higher positive affect and longevity did not differ as a function of perceived stress in participants without any chronic conditions and participants with diabetes. However, in a subsample of participants over the age of 65 with no chronic conditions, the positive association between positive affect and longevity was strongest among participants that reported higher stress. There are differences between our study and the study by Moskowitz et al. [29] that might account for these divergent findings. First, the larger sample size (n = 8542 vs. 2890) in our study may have increased our chance of detecting an interaction effect. Second, Moskowitz et al. [29] used the positive affect subscale from the CES-D and we used the positive affect subscale from the GWQ. This may have made a difference because the CES-D subscale differs from the GWQ in that it contains questions regarding self-esteem and hope for the future (as well as happiness and enjoyment). In supplementary Cox regressions where we replaced the GWQ positive affect measure with the CES-D subscale, we found that the interaction between perceived stress and positive affect was significant in the age- and sex-adjusted model (p = 0.017) but not in the model additionally adjusted for demographic factors (p = 0.065) or the fully adjusted model (p = 0.078). It is possible that the type of positive affect measured in the GWQ (feeling in high spirits, happy, and full of energy) plays a greater role in buffering against the deleterious effects of stress.

There is evidence that the subdomain of energy/vitality may underlie associations between higher positive affect and longevity [1]. To test whether this is the case in these data, we repeated the main analysis excluding the vitality item from the GWQ positive affect measure. The interaction between perceived stress and positive affect in the fully adjusted model was not significant. This finding suggests that the vitality subdomain may have partially driven the negative association between positive affect and mortality risk. Previous longitudinal studies have documented an association between higher emotional vitality and lower risk of cardiovascular disease death [76] or death from all-causes [77]. Kubzansky and Thurnstone [76] suggested that, as well as dampening physiological responses to stress, high emotional vitality may confer cognitive (e.g., concentration or problem solving) and social advantages that help protect against mortality risk.

According to the stress-buffering model, the experience of stress negatively impacts health. This prediction was only partially supported by our results. We found a positive association between stress and mortality risk in an age- and sex-adjusted model. This association was not significant following adjustment for demographic differences, depressive symptoms, history of chronic disease and health behaviours—suggesting that these factors may account for the positive link between stress and mortality risk. Although the positive association between stress and risk of mortality from cardiovascular disease is relatively well established [66], findings regarding the association between stress and all-cause mortality have been mixed. In a sample of 12,128 Danish participants, following adjustment for established risk factors, men with high stress had a higher risk of mortality; however, there was no association between stress and all-cause mortality risk among women [67]. In a study of 4132 Taiwanese older adults, the positive association between perceived stress and risk of all-cause mortality was not significant following adjustment for depressive symptoms, mobility limitations and medical conditions [68]. Surprisingly, in our study, the relationship between stress and mortality risk became inverse and significant following additional adjustment for positive affect. It is unclear why this was the case. However, as low positive affect was associated with a higher mortality risk and participants with higher perceived stress reported lower positive affect, it is possible that positive affect partially confounded the positive association between perceived stress and mortality risk. Although reports of stress have generally been linked with poorer health outcomes, there is evidence that the experience of (short-term) moderate stress can be beneficial [69]. Liu and Vickers [69] suggest that the experience of moderate stress may help individuals become more resilient. High resilience has been linked to favourable health outcomes [70], [71].

The temporal relationship between positive affect and perceived stress was unclear in our study as participants were asked to report the degree to which they experienced positive affect and stress within the past month. Positive affect could have preceded, followed or co-occurred with the experience of stress. Further work is needed to investigate the relationship between positive affect and physical health at each of these time points. Notably, although perceived stress and positive affect were assessed at one time point in our study, the interaction between these variables in predicting mortality risk was apparent over the 10-year follow-up period. This suggests that our findings reflect relatively stable (i.e., trait) differences in perceived stress and positive affect. Previous work shows that positive affect is closely related to personality (specifically, extraversion) [72] and can remain stable even over long periods of time (20 years) [73]. To explore this further, we examined the stability of positive affect and perceived stress measures between NHANES 1 and NHEFS (1982) (perceived stress and positive affect were not measured in subsequent waves of the NHEFS). Surprisingly, the measures were only moderately stable; the test-retest reliability was 0.43 for positive affect and 0.42 for perceived stress.

The stress measure in our study was subjective rather than objective. Holme and Rahe's [74] Social Readjustment Rating Scale—which requires participants to indicate the number of pre-defined stressful events they have experienced—could provide a more objective alternative. However, previous work indicates that, compared with stressful life event measures, subjective measures of stress—that are sensitive to individual differences in appraisal—are more strongly related to mental and physical health [75]. It should also be noted that the subjective stress measure in our study was strongly positively correlated with depressive symptoms and strongly negatively correlated with positive affect.

Our study had several strengths, including the use of a large nationally representative sample, the fact that mortality data were obtained from death certificates rather than by proxy reports, and the availability of many measures that enabled us to control for many potential confounds. One limitation of our study was that a substantial proportion of participants were excluded due to missing data. This may have introduced a source of bias as excluded participants differed from included participants on several covariates (see Supplementary Table 1). In addition, our perceived stress measure was not sensitive to the cause or duration of stress. Both of these factors affect the strength of association between stress and physical health and warrant consideration in future studies [14].

## 5 Conclusion

Our findings indicate that the positive association between positive affect and longevity may not be universal, but depend on perceived stress, and possibly other psychosocial processes. Research concerning links between positive affect and mortality risk should test for the presence of stress buffering mechanisms. On a practical note, authors have proposed that interventions designed to increase positive affect may promote health among older adults [3], [78]. Our results indicate that such interventions may be most effective among groups who report high levels of stress.

## Sources of funding

J.A. Okely and C.R. Gale are members of The University of Edinburgh Centre for Cognitive Ageing and Cognitive Epidemiology, part of the cross council Lifelong Health and Wellbeing Initiative (MR/K026992/1). Funding from the Biotechnology and Biological Sciences Research Council and Medical Research Council is gratefully acknowledged.

## Competing interests

All authors have completed the Unified Competing Interest form and declare they have no competing interests to report.

## Appendix A Supplementary data

Supplementary tables Image 1
