# Supplementary material for: The interaction between stress and positive affect in predicting mortality
Source: J Psychosom Res. 2017 Sep;100:53–60. doi: 10.1016/j.jpsychores.2017.07.005 (PMC5555349; doi:10.1016/j.jpsychores.2017.07.005)
Supplement: Supplementary file 1 — Supplementary tables [file mmc1.docx]

| Supplementary Table 1  *Baseline characteristics of participants included and excluded in the analytic sample* | | | | |
| --- | --- | --- | --- | --- |
| **Characteristic** | **Included** | **Excluded** | **Total N^a^** | **p-trend^b^** |
| Positive affect score *M* (SD) | 13 (4) | 12 (4) | 9,974 | <0.001 |
| CESD score *Mdn* (IQR) | 8 (2-12) | 10 (3-14) | 9,512 | <0.001 |
| Stress score *M* (SD) | 8 (4) | 8 (5) | 9,969 | 0.001 |
| Age *M* (SD) | 55.89 (14.47) | 62.14 (15.46) | 10,523 | <0.001 |
| BMI (kg/m2) *M* (SD) | 26.30 (4.97) | 26.03 (5.11) | 9,972 | 0.066 |
| Female, No. (%) | 5371 (63) | 1907 (52) | 12,220 | <0.001 |
| Race/Ethnicity No. (%) |  |  | 12,220 | <0.001 |
| Black | 1032 (12) | 670 (18) |  |  |
| White | 7425 (87) | 2974 (81) |  |  |
| Other | 85 (1) | 34 (1) |  |  |
| Married, No. (%) | 5953 (70) | 2110 (58) | 12,181 | <0.001 |
| Wealth category, No. $ (%) |  |  | 11,789 | <0.001 |
| <3,000 | 887 (10) | 809 (25) |  |  |
| 3,000-5,999 | 1385 (16) | 753 (23) |  |  |
| 6,000-14,999 | 4246 (50) | 1240 (38) |  |  |
| >14,999 | 2024 (24) | 445 (14) |  |  |
| Education category, No. (%) |  |  | 12,146 | <0.001 |
| ≤8 years | 1761(21) | 1501(42) |  |  |
| 9-11 years | 1403 (16) | 634 (18) |  |  |
| 12 years | 3188 (37) | 872 (24) |  |  |
| >12 years | 2190 (26) | 597 (17) |  |  |
| Recreational activity, No. (%) |  |  | 10,063 | <0.001 |
| Inactive | 2915 (34) | 657 (43) |  |  |
| Moderate | 4248 (50) | 651 (43) |  |  |
| Vigorous | 1379 (16) | 213 (14) |  |  |
| Non-recreational activity, No. (%) |  |  | 10,063 | <0.001 |
| Inactive | 1395 (16) | 337 (22) |  |  |
| Moderate | 4792 (56) | 796 (52) |  |  |
| Vigorous | 2355 (28) | 385 (25) |  |  |
| Alcohol consumption, No. (%) |  |  | 12,170 | <0.001 |
| Abstainer | 3688 (43) | 2712 (75) |  |  |
| Light drinker | 3890 (46) | 730 (20) |  |  |
| Moderate drinker | 964 (11) | 186 (5) |  |  |
| Smoking status, No. (%) |  |  | 11,185 | <0.001 |
| Non smoker | 3873 (45) | 1609 (61) |  |  |
| Former smoker | 2287 (27) | 538 (20) |  |  |
| Smoker | 2382 (28) | 496 (19) |  |  |
| ≥ 5 fruit and vegetables, No. (%) | 3162 (37) | 663 (38) | 10,298 | 0.577 |
| Sleep duration categories, No. (%) |  |  | 9,789 | <0.001 |
| <5 hours | 206 (2) | 65 (5) |  |  |
| 5-9 hours | 8076 (95) | 1107 (89) |  |  |
| >9 hours | 260 (3) | 75 (6) |  |  |
| History of CVD, No. (%) | 507 (6) | 924 (28) | 11,833 | <0.001 |
| History of cancer, No. (%) | 749 (9) | 714 (20) | 12,154 | <0.001 |
| History of chronic lung disease, No. (%) | 860 (10) | 516 (14) | 12171 | <0.001 |
| ^a^ number of participants with data  ^b^ statistical significance is based χ2 tests or t-tests, as appropriate. | | | | |

| Supplementary Table 2.  *Correlations Among Predictor and Covariate Variables* | | | | | | | | | | | | | | | | | | |  |
| --- | --- | --- | --- | --- | --- | --- | --- | --- | --- | --- | --- | --- | --- | --- | --- | --- | --- | --- | --- |
| **Variable** | **1** | | **2** | **3** | **4** | **5** | **6** | **7** | **8** | **9** | **10** | **11** | **12** | **13** | **14** | **15** | **16** | **17** | **18** |
| 1 Stress | | - | .56 | -.51 | -.20 | .00 | -.14 | .00 | -.03 | .03 | .08 | -.11 | .03 | .04 | .00 | -.09 | .04 | -.02 | .08 |
| 2 CESD | | | - | -.54 | .05 | .03 | -.11 | -.14 | .07 | -.16 | -.17 | -.17 | -.08 | .02 | -.07 | -.07 | .08 | .02 | .09 |
| 3 Positive affect | | | | - | -.12 | -.04 | .11 | .13 | -.01 | .17 | .14 | .29 | .09 | -.01 | .05 | .04 | -.12 | -.04 | -.14 |
| 4 Age | | | | | - | -.04 | .09 | -.22 | -.01 | -.30 | -.35 | -.14 | -.23 | -.19 | .04 | .07 | .19 | .19 | .09 |
| 5 BMI | | | | | | - | .02 | .00 | .11 | -.05 | -.09 | -.13 | -.07 | -.09 | -.01 | -.03 | .00 | -.03 | -.03 |
| 6 Sex | | | | | | | - | .02 | -.04 | .09 | -.01 | .11 | .20 | .17 | -.10 | .03 | .09 | .00 | .01 |
| 7 Married | | | | | | | | - | -.13 | .35 | .17 | .09 | .08 | .02 | .05 | -.01 | -.02 | -.03 | -.04 |
| 8 Ethnicity | | | | | | | | | - | -.18 | -.12 | -.06 | -.08 | .02 | -.09 | -.02 | -.02 | -.07 | -.04 |
| 9 Wealth | | | | | | | | | | - | .46 | .12 | .26 | .08 | .06 | -.01 | -.09 | -.04 | -.06 |
| 10 Education | | | | | | | | | | | - | .12 | .26 | .01 | .09 | -.02 | -.12 | -.03 | -.09 |
| 11 Exercise | | | | | | | | | | | | - | .10 | .03 | .03 | -.01 | -.10 | -.03 | -.07 |
| 12 Alcohol | | | | | | | | | | | | | - | .29 | -.07 | -.01 | -.06 | -.04 | -.03 |
| 13 Smoking | | | | | | | | | | | | | | - | -.11 | -.01 | 0.02 | -.03 | .11 |
| 14 Diet | | | | | | | | | | | | | | | - | .02 | .01 | .01 | -.01 |
| 15 Sleep | | | | | | | | | | | | | | | | - | .00 | .03 | -.01 |
| 16 CVD | | | | | | | | | | | | | | | | | - | .04 | .08 |
| 17 Cancer | | | | | | | | | | | | | | | | | | - | .04 |
| 18 Lung disease | | | | | | | | | | | | | | | | | | | - |
| Higher scores = better diet, more years education, more exercise, greater alcohol consumption, being female, being married and having a history of chronic disease.  Smoking coded as: current = 3, former = 2, non-smoker = 1.  Race/ethnicity coded as: other = 3, black = 2, white = 1. | | | | | | | | | | | | | | | | | | | |

| Supplementary Table 3  *Bivariate Associations for Positive Affect, Stress and Covariate Variables with Mortality Risk* | | |
| --- | --- | --- |
| **Variable** | **HR (95% CI)** | ***p*** |
| Positive affect | 0.73 (0.69-0.76) | <0.001 |
| CESD | 1.02 (1.02-1.03) | <0.001 |
| Stress | 0.83 (0.78-0.87) | <0.001 |
| Age | 1.10 (1.09-1.10) | <0.001 |
| BMI | 0.97 (0.96-0.98) | <0.001 |
| Sex: male vs. female | 1.91 (1.72-2.11) | <0.001 |
| Race/Ethnicity |  |  |
| Black vs. white | 1.22 (1.05-1.41) | 0.008 |
| Other ethnicity vs. white | 0.90 (0.52-1.56) | 0.72 |
| Wealth $ |  |  |
| 3,000-5,999 vs. <3,000 | 0.67 (0.58-0.78) | <0.001 |
| 6,000-14,999 vs. <3,000 | 0.33 (0.28-0.37) | <0.001 |
| >14,999 vs. <3,000 | 0.21 (0.17-0.25) | <0.001 |
| Education |  |  |
| 9-11 years vs. ≤8 years | 0.44 (0.38-0.51) | <0.001 |
| 12 years vs. ≤8 years | 0.25 (0.22-0.29) | <0.001 |
| >12 years vs. ≤8 years | 0.26 (0.23-0.30) | <0.001 |
| Recreational activity |  |  |
| Moderate vs. inactive | 0.56 (0.51-0.63) | <0.001 |
| Vigorous vs. inactive | 0.41 (0.35-0.49) | <0.001 |
| Non-recreational activity |  |  |
| Moderate vs. inactive | 0.50 (0.45-0.57) | <0.001 |
| Vigorous vs. inactive | 0.32 (0.28-0.38) | <0.001 |
| Alcohol consumption |  |  |
| Light drinker vs. abstainer | 0.58 (0.52-0.65) | <0.001 |
| Moderate drinker vs. abstainer | 0.57 (0.47-0.68) | <0.001 |
| Smoking status |  |  |
| Former smoker vs. non smoker | 1.31 (1.17-1.48) | <0.001 |
| Smoker vs. non smoker | 0.89 (0.79-1.02) | 0.086 |
| Diet: ≥ 5 fruit and vegetables vs. < 5 | 0.87 (0.78-0.97) | 0.012 |
| Sleep duration |  |  |
| 5-9 hours vs. <5 hours | 0.58 (0.44-0.76) | <0.001 |
| >9 hours vs. <5 hours | 1.58 (1.14-2.19) | 0.006 |
| History of CVD vs. no history | 4.02 (3.51-4.61) | <0.001 |
| History of cancer vs. no history | 2.60 (2.28-2.97) | <0.001 |
| History of chronic lung disease vs. no history | 2.05 (1.79-2.34) | <0.001 |

| Supplementary Table 4  *HRs (95% CIs) for all-cause mortality according to a SD increase in positive affect score with and without the vitality item divided by tertiles of stress score* | | | |
| --- | --- | --- | --- |
| **Model** | **Stress tertile** | **With vitality item** | **Without vitality item** |
| Model 1 | Low | 0.87 (0.80-0.94)** | 0.93 (0.85-1.02) |
|  | Moderate | 0.76 (0.67-0.86)** | 0.88 (0.76-0.97)* |
|  | High | 0.69 (0.63-0.76)** | 0.75 (0.68-0.83)** |
| Model 2 | Low | 0.90 (0.82-0.98)* | 0.97 (0.89-1.07) |
|  | Moderate | 0.82 (0.72-0.93)** | 0.95 (0.83-1.09) |
|  | High | 0.77 (0.68-0.86)** | 0.86 (0.76-0.97)* |
| Model 3 | Low | 0.98 (0.89-1.08) | 1.01 (0.91-1.11) |
|  | Moderate | 0.85 (0.74-0.97)* | 0.97 (0.85-1.11) |
|  | High | 0.84 (0.75-0.95)** | 0.92 (0.82-1.04) |
| Model 1: Adjusted for age and sex. Model 2 Further adjusted for demographic factors, history of chronic disease and depressive symptoms. Model 3 additionally adjusted for health behaviors, sleep duration and BMI. ** *p* <0.001 * *p* <0.05 | | | |
